# Molecular Antimicrobial Resistance Surveillance for *Neisseria gonorrhoeae*, Northern Territory, Australia 

**DOI:** 10.3201/eid2309.170427

**Published:** 2017-09

**Authors:** David M. Whiley, Ella Trembizki, Cameron Buckley, Kevin Freeman, Robert W. Baird, Miles Beaman, Marcus Chen, Basil Donovan, Ratan L. Kundu, Christopher K. Fairley, Rebecca Guy, Tiffany Hogan, John M. Kaldor, Mahdad Karimi, Athena Limnios, David G. Regan, Nathan Ryder, Jiunn-Yih Su, James Ward, Monica M. Lahra

**Affiliations:** Pathology Queensland Central Laboratory, Brisbane, Queensland, Australia (D. Whiley);; The University of Queensland, Brisbane (D. Whiley, E. Trembizki, C. Buckley);; Royal Darwin Hospital, Darwin, Northern Territory, Australia (K. Freeman, R.W. Baird);; University of Western Australia, Nedlands, Western Australia, Australia (M. Beaman);; University of Notre Dame Australia, Fremantle, Western Australia, Australia (M. Beaman);; Western Diagnostic Pathology, Perth, Western Australia, Australia (M. Beaman, M. Karimi);; Alfred Health, Carlton, Victoria, Australia (M. Chen, C.K. Fairley);; Central Clinical School, Monash University, Melbourne, Victoria, Australia (M. Chen, C.K. Fairley);; Sydney Hospital, Sydney, New South Wales, Australia (B. Donovan);; University of New South Wales, Sydney (B. Donovan, R. Guy, J.M. Kaldor, D.G. Regan, N. Ryder, M.M. Lahra);; Prince of Wales Hospital, Sydney (R.L. Kundu, T. Hogan, A. Limnios, M.M. Lahra);; School of Medical Sciences, Hunter New England Local Health District, Sydney (N. Ryder);; Menzies School of Health Research and Charles Darwin University, Darwin (J.-Y. Su);; South Australian Health and Medical Research Institute, Adelaide, South Australia, Australia (J. Ward);; Flinders University, Adelaide (J. Ward)

**Keywords:** *Neisseria gonorrhoeae*, antimicrobial resistance, molecular, surveillance, gonococcus, gonorrhea, bacteria, culture, Australia, Northern Territory, antimicrobial drugs, AMR

## Abstract

*Neisseria gonorrhoeae* antimicrobial resistance (AMR) is a globally recognized health threat; new strategies are needed to enhance AMR surveillance. The Northern Territory of Australia is unique in that 2 different first-line therapies, based primarily on geographic location, are used for gonorrhea treatment. We tested 1,629 *N. gonorrhoeae* nucleic acid amplification test–positive clinical samples, collected from regions where ceftriaxone plus azithromycin or amoxicillin plus azithromycin are recommended first-line treatments, by using 8 *N. gonorrhoeae* AMR PCR assays. We compared results with those from routine culture-based surveillance data. PCR data confirmed an absence of ceftriaxone resistance and a low level of azithromycin resistance (0.2%), and that penicillin resistance was <5% in amoxicillin plus azithromycin regions. Rates of ciprofloxacin resistance and penicillinase-producing *N. gonorrhoeae* were lower when molecular methods were used. Molecular methods to detect *N. gonorrhoeae* AMR can increase the evidence base for treatment guidelines, particularly in settings where culture-based surveillance is limited.

Resistance to antimicrobial drugs (termed antimicrobial resistance [AMR]) in *Neisseria gonorrhoeae* is recognized as a public health problem of global importance (*1**,**2*). The overall magnitude of AMR in *N. gonorrhoeae* is largely unknown in many regions because of substantial gaps in global AMR surveillance (*3*). Ceftriaxone, a third-generation extended-spectrum cephalosporin used widely for treatment, is considered to be the last fully effective option currently recommended. However, its durability is not assured; the proportion of gonococcal strains with elevated ceftriaxone MIC values is increasing steadily, and 4 documented cases of resistance to ceftriaxone have been noted, in Japan, France, Spain, and Australia (*4**–**6*)*.* A dual therapy treatment regimen of ceftriaxone plus azithromycin is now the recommended standard of care in many countries and was implemented in an effort to stem further development of ceftriaxone resistance (*7*); however, failure of this dual therapy has also been reported (*8*).

Surveillance of *N. gonorrhoeae* antimicrobial drug sensitivity is a key component of all strategies to manage the emergence of resistance; gonorrhea treatment guidelines are currently formulated under the principle that an antimicrobial drug should be rejected for clinical use in a particular population once a threshold of 5% resistant isolates is breached (*1*). However, although gonorrhea diagnosis is now widely based on nucleic acid methods, antimicrobial drug sensitivity remains dependent on culture-based methods, which are highly specific but difficult to implement in many settings where gonorrhea is prevalent. The World Health Organization (WHO) and Centers for Disease Control and Prevention (CDC), in their action plans to control the spread of drug-resistant *N. gonorrhoeae*, have called for the development of molecular assays (*1**,**2*). We have previously reviewed the potential for real-time PCR assays to achieve these goals (*3*) and, through the Gonorrhoea Resistance Assessment by Nucleic Acid Detection (GRAND) study, developed and validated a range of PCR methods to detect resistance mutations for azithromycin, ciprofloxacin, and penicillin. We have also developed a high-throughput single-nucleotide polymorphism–based typing method for multilocus sequence type (MLST) investigations (*9*).

In Australia, gonorrhea is concentrated primarily in men who have sex with men living predominantly in urban and regional areas and in Aboriginal heterosexual persons living in remote areas of central and northern Australia. Dual therapy comprising ceftriaxone (500 mg intramuscular injection) and oral azithromycin (1 g) (known as CAZ) is used as the standard first-line treatment in urban areas. However, in remote areas of the Northern Territory (Figure 1) and Western Australia, where rates of infection among Aboriginal populations are high and AMR is low, first-line treatment is oral amoxicillin 3 g, probenecid 1 g, and azithromycin 1 g (locally known as a ZAP pack), a treatment strategy that has the benefits of oral administration and avoidance of the unnecessary use of ceftriaxone.

**Figure 1:**
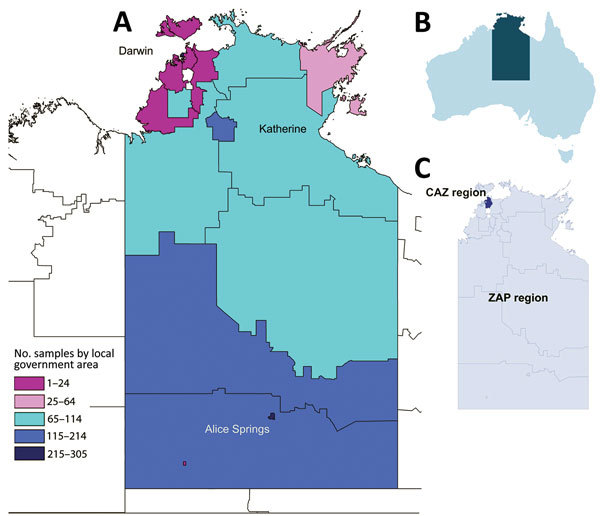
Collection areas for *Neisseria gonorrhoeae* samples within the Northern Territory of Australia, 2014. A) Heat map showing the local government areas from which the 1,629 nucleic acid amplification test–positive clinical samples were collected. B) Location of the Northern Territory within Australia. C) Location of the CAZ and ZAP regions within the Northern Territory. CAZ, ceftriaxone via intramuscular injection and oral azithromycin; ZAP, azithromycin, amoxicillin, probenecid.

In remote parts of Australia, including much of the ZAP pack region, transport distances and climate pose limits on the viability of samples for culture. Also, even though all clinicians diagnosing or suspecting gonorrhea in Australia are encouraged to send a suitable swab specimen for AMR testing by culture, changes to test reimbursement schemes have tended to favor use of nucleic acid amplification test (NAAT) over culture for gonorrhea diagnosis. For these combined reasons, relatively few isolates are available for AMR testing from these areas (*10*). Our PCR-based method (*11**,**12*) for direct detection of penicillinase-producing *N. gonorrhoeae* (PPNG) is now routinely used to help inform treatment guidelines in Western Australia (*13*) and the Northern Territory. We report on a study that we have undertaken in the Northern Territory to comprehensively examine the utility of nucleic acid methods to assess AMR against a broad range of antimicrobial drugs, and the potential role of these methods in setting treatment guidelines. The study was approved by the Human Research Ethics Committee (HREC) of Northern Territory Department of Health and Menzies School of Health, the Central Australian HREC, the South Eastern Sydney Local Health District HREC, the Queensland Children’s Health Services District HREC, and the University of Queensland HREC.

## Material and Methods

### *N. gonorrhoeae* NAAT-Positive Clinical Samples

For this study, we examined 1,629 *N. gonorrhoeae* NAAT-positive clinical samples collected from patients in the Northern Territory (441 from CAZ regions and 1,188 from ZAP regions; Figure 1) during 2014; these samples represented 93.6% of the total 1,740 notifications of gonorrhea in this region for this year. The samples comprised 21 rectal, 45 pharyngeal, 413 vaginal/cervical, 97 male urethral, and 1 ocular specimen, as well as 1,012 urine samples (590 from men, 418 from women, and 4 from persons whose sex was not specified), 21 joint fluid samples, and 19 samples for which the site was not recorded on the request form. Samples were taken from 756 (46.4%) men, 861 (52.9%) women, and 12 (0.7%) persons for whom sex was not specified; median age was 24 years. The specimens were provided by the 2 laboratories in the Northern Territory that diagnose gonorrhea: Royal Darwin Hospital Pathology, which used Siemens Versant *Chlamydia trachomatis*/*N. gonorrhoeae* (CT/NG) assays (Siemens, Bayswater, Victoria, Australia), and Western Diagnostic Pathology, which used the Aptima Combo 2 test for CT/NG (Hologic, Bedford, MA, USA) (additional information in Technical Appendix Table). 

### PCR Methods 

We used 8 PCR methods for AMR testing (Table 1): assays to detect PPNG (PPNG-PCR) (*11*); the mosaic penicillin binding protein 2 (PBP2) associated with resistance to the extended-spectrum cephalosporins (mosaicPBP2-PCR); the A8806 strain of ceftriaxone-resistant gonorrhea previously detected in Australia (A8806-PCR assay) (*6*); the GyrA S91F alteration that is highly predictive of resistance to ciprofloxacin (GyrA91-PCR) (*14**,**15*); 2 mutations on the 23S rRNA genes conferring resistance to azithromycin (2611-PCR and 2059-PCR) (*16*); wild-type PorB sequences (G101/A102 and PorB1a) that we have shown are predictive of penicillin susceptibility, and mutant PorB sequences (G101K/A102D, G101K/A102N and G101K/A102G) that are associated with but not predictive of penicillin resistance (PorB-PCR) (*17*); and a wild-type *mtrR* efflux pump promoter (i.e., lacking the A-deletion) shown to be associated with susceptibility to β lactam antimicrobial drugs (*mtrR* A-deletion-PCR) (Technical Appendix; *18*). We considered samples uncharacterized by a method if they did not provide a result (e.g., of mutant or wild type) in the respective PCR. For mosaicPBP2-PCR and A8806-PCR, we pooled samples (10 samples/pool) for testing; we then tested samples from any pools returning positive results individually. For all other PCR methods, we tested samples individually (Technical Appendix). 

**Table 1:** PCR results for the 1,629 NAAT-positive *Neisseria gonorrhoeae* clinical samples taken from patients in the Northern Territory of Australia, 2014*

Assay and targets	Results							
CAZ region, n = 441			ZAP region, n = 1,188			Total for both regions, n = 1,629	
No. samples/no. tested	% Samples (95% CI)		No. samples/no. tested	% Samples (95% CI)		No. samples/no. tested	% Samples (95% CI)
GyrA91-PCR (*gyrA*)								
Wild type (S91)	235/297	79.1 (74.1–83.4)		922/931	99.0 (98.2–99.5)		1,157/1,228	94.2 (92.8–95.4)
S91F	62/297	20.9 (16.6–25.9)		9/931	1.0 (0.5–1.8)		71/1,228	5.8 (4.6–7.2)
Uncharacterized†	144	32.6		257	21.6		401/1,629	24.6
PPNG-PCR								
Negative	252/286	88.1 (83.8–91.4)		879/888	99 (98.1–99.5)		1,131/1,174	96.3 (95.1–97.3)
PPNG	34/286	11.9 (8.6–16.2)		9/888	1 (0.5–1.9)		43/1,174	3.7 (2.7–4.9)
Uncharacterized†	155	35.1		300	25.3		455/1,629	27.9
2611-PCR (23S rRNA)								
Wild type	321/321	100 (98.8–100)		958/960	99.8 (99.2–99.9)		1,279/1,281	99.8 (99.4–100)
C2611T	0/321	0 (0–1.2)		2/960	0.2 (0.1–0.8)		2/1,281	0.2 (0–0.6)
Uncharacterized†	120	27.2		228	19.2		348/1,629	21.4
2059-PCR (23S rRNA)								
Wild type	319/319	100 (98.8–100)		1,004/1,004	100 (99.6–100)		1,323/1,323	100 (99.7–100)
A2059G	0/319	0 (0–1.2)		0/1,004	0 (0–0.4)		0/1,323	0 (0–0.3)
Uncharacterized†	122	27.7		184	15.5		306/1,629	18.8
PorB-PCR (*porB*)								
G101/A102	98/286	34.3 (29–39.9)		125/835	15.0 (12.7–17.6)		223/1,121	19.9 (17.7–22.3)
[G101/A102, non-PPNG]‡	[80/280]	[28.6 (23.4–34.1)]		[116/823]	[14.1 (11.9–16.6)]		[196/1,103]	[17.8 (15.6–20.1)]
PorB1a	152/286	53.1 (47.4–58.9)		699/835	83.7 (81.1–86.1)		851/1,121	75.9 (73.3–78.3)
[PorB1a, non-PPNG]‡	[120/267]	[44.9 (39.1–50.9)]		[627/770]	[81.3 (78.4–83.9)]		[747/1,037]	[72.0 (69.2–74.7)]
Mutant§	32/286	11.2 (8–15.4)		3/835	0.4 (0.1–1.1)		35/1,121	3.1 (2.3–4.3)
PorB1a and G101/A102	4/286	1.4 (0.5–3.5)		6/835	0.7 (0.3–1.6)		10/1,121	0.9 (0.5–1.6)
PorB1a and mutant§	0/286	0 (0–1.3)		2/835	0.2 (0.1–0.9)		2/1,121	0.2 (0–0.6)
Uncharacterized†	155	35.1		353	29.8		508/1,629	31.2
A-deletion-PCR (*MtrR*)								
Wild type	280/318	88.1 (84–91.2)		944/950	99.4 (98.6–99.7)		1,224/1,268	96.5 (95.4–97.4)
[Wild type, non-PPNG]‡	[226/284]	[79.6 (74.5–84.1)]		[861/873]	[98.6 (97.6–99.2)]		[1,087/1,157]	[93.9 (92.4–95.2)]
A-deletion	38/318	11.9 (8.8–16)		6/950	0.6 (0.3–1.4)		44/1268	3.5 (2.6–4.6)
Uncharacterized†	123	27.9		238	20		361/1629	22.2
mosaicPBP2-PCR (*penA*)								
Negative	431/441	97.7 (95.9–98.8)		1,188/1,188	100 (99.7–100)		1,619/1,629	99.4 (98.9–99.7)
Positive	10/441	2.3 (1.2–4.1)		0/1188	0 (0–0.3)		10/1,629	0.6 (0.3–1.1)
Uncharacterized†¶	NA	NA		NA	NA		NA	NA
A8806-PCR (*penA*)								
Negative	441/441	100 (99.1–100)		1,188/1,188	100 (99.7–100)		1,629/1,629	100 (99.8–100)
Positive	0/441	0 (0–0.9)		0/1,188	0 (0–0.3)		0/1,629	0 (0–0.2)
Uncharacterized†¶	NA	NA		NA	NA		NA	NA

*NA, not applicable; NAAT, nucleic acid amplification test; PPNG, penicillinase-producing *N. gonorrhoeae*. †These samples could not be characterized by the respective PCR assay.  ‡These PorB-PCR and A-deletion-PCR results were combined with the PPNG-PCR results to exclude those that were PPNG. Uncharacterized PPNG-PCR results were omitted from total numbers. §Mutant = G101K/A102D, G101K/A102N or G101K/A102G.  ¶All negative results for the mosaicPBP2-PCR and A8806-PCR were considered valid (i.e., characterized) given that pooled samples were used for screening, and it was therefore not possible to ascertain characterization for individual samples.

### MLST

We genotyped the first 717 samples (collected during January–May 2014) using a previously described iPLEX14SNP method (*9*). The iPLEX14SNP targets 14 informative single-nucleotide polymorphisms on gonococcal housekeeping genes and provides a high-throughput means of conducting *N. gonorrhoeae* MLST. We further used these data to clarify the potential movement of strains between the CAZ and ZAP zones, irrespective of their AMR profiles.

### Multiantigen Sequence Typing

We further investigated samples testing positive for mosaic PBP2 by using *N. gonorrhoeae* multiantigen sequence typing (NG-MAST) (*19*) as described previously (*20*). NG-MAST involves DNA sequencing of 2 variable gonococcal genes and therefore provides greater discrimination than MLST.

### Australian Gonococcal Surveillance Program Data

The Australian Gonococcal Surveillance Program receives and tests clinical isolates of gonococci for the purposes of AMR phenotypic surveillance from throughout Australia. In 2014, the program received 222 isolates from the Northern Territory for antimicrobial drug susceptibility testing (Table 2). We compared the phenotypic rates of resistance with the results of PCR testing for the Northern Territory as a whole and individually by CAZ and ZAP regions.

**Table 2:** Summary of the Australian Gonococcal Surveillance Program culture-based *Neisseria gonorrhoeae* antimicrobial drug resistance data for the Northern Territory of Australia, 2014*

Antimicrobial drug	CAZ region, n = 81			ZAP region, n = 141			Total, n = 222	
No. isolates	% Isolates (95% CI)	No. isolates	% Isolates (95% CI)	No. isolates	% Isolates (95% CI)
Azithromycin R	0	0.0 (0.0–4.5)		0	0.0 (0.0–2.7)		0	0.0 (0.0–1.7)
Ceftriaxone DS	3	3.7 (1.3–10.3)		0	0.0 (0.0–2.7)		3	1.4 (0.5–3.9)
Ciprofloxacin R	25	30.9 (21.9–41.6)		3	2.1 (0.7–6.1)		28	12.6 (8.9–17.6)
Penicillin R	21	25.9 (17.6–36.4)		3	2.1 (0.7–6.1)		24	10.8 (7.4–15.6)
PPNG	15	18.5 (11.6–28.3)		3	2.1 (0.7–6.1)		18	8.1 (5.2–12.5)

*Adapted from the Australian Gonococcal Surveillance Program annual report) (*10*). DS, decreased susceptibility; PPNG, penicillinase-producing *N. gonorrhoeae*; R, resistant.

## Results

Of the 1,629 samples, 18.8% to 31.2% could not be characterized by the PCR methods (Table 1). However, these values excluded the mosaicPBP2-PCR and A8806-PCR methods; we considered all negative results for the mosaicPBP2-PCR and A8806-PCR characterized because pooled samples were used for screening, and it was therefore not possible to ascertain characterization for individual samples. Compared with culture, which provided 81 isolates from CAZ areas and 141 from ZAP regions in this year (Table 2), the PCR assays (based on the average number of characterized samples by each PCR) provided 4.2 times more data (mean 338.6 samples) from CAZ regions and 9.4 times more data (mean 1,331.6 samples) from ZAP regions.

We compared characterization rates based on anatomic site and originating laboratory (Technical Appendix Table); only the GyrA91-PCR results were used for this comparison. We observed a significantly lower (p<0.001) characterization rate for pharyngeal samples (26.7%) compared with the combined rate (76.7%, range 56.7%–100%) for all other sample types.

### *N. gonorrhoeae* Resistance

We compared PCR (Table 1) and culture (Table 2) data for resistance to azithromycin, ceftriaxone, ciprofloxacin, and penicillin. The combined results of the 2611-PCR and 2059-PCR assays showed that *N. gonorrhoeae* azithromycin resistance was low (0.2%) across the Northern Territory in 2014, consistent with the 2014 culture-based data that showed no azithromycin-resistant isolates. We found 10 (0.6%) samples positive by the mosaicPBP2-PCR, all in CAZ regions; these data were consistent with 3 (1.4%) isolates exhibiting decreased susceptibility to ceftriaxone in CAZ regions detected by culture. We observed no A8806-PCR positive samples, consistent with culture data showing no *N. gonorrhoeae* with MICs for ceftriaxone >0.125 mg/L isolated in the Northern Territory or elsewhere in Australia since the A8806 strain was observed in late 2013. 

Total ciprofloxacin resistance in the Northern Territory was significantly lower by PCR (5.8%) than by bacterial culture (12.6%; p<0.001). Consistent with the culture data, the GyrA91-PCR indicated that levels of ciprofloxacin resistance were higher in the CAZ region compared with the ZAP region. Similarly, total levels of PPNG in the Northern Territory as determined by PCR (3.7%) were significantly lower than the levels determined by culture (8.1%); p<0.01). For ZAP regions, PPNG-PCR testing showed that 1.0% of samples were PPNG positive, comparable to the 2.1% observed with bacterial culture. 

Using bacterial culture, we detected no isolates with chromosomally mediated resistance to penicillin (CMRP) in ZAP regions. Although the porB-PCR is not a definitive marker of CMRP (*17*), it was still able to provide insight into CMRP, given that the G101/A102 and PorB1a markers are highly predictive of penicillin susceptibility (in which PPNG strains are simultaneously detected and excluded). In the ZAP regions, 14.1% of samples were both non-PPNG and G101/A102 and 81.3% were both non-PPNG and PorB1a, providing 95.4% of samples predicted to be susceptible to penicillin. Thus, based on the combined porB-PCR and PPNG-PCR results, we predicted by PCR that 95.4%–99% of samples were susceptible to penicillin. These data are further supported by the combined A-deletion-PCR and PPNG-PCR data; 98.6% of samples from ZAP regions possessed a wild-type *mtrR* promoter and were non-PPNG.

### MLST

**Figure 2:**
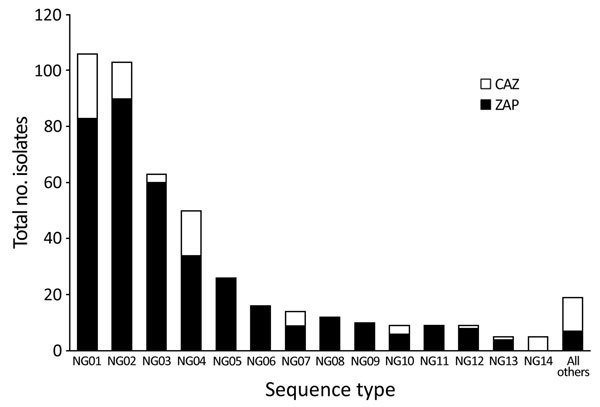
Genotype frequency of the 456 *Neisseria gonorrhoeae* clinical samples taken from patients in the Northern Territory of Australia, 2014, that were successfully genotyped by using the iPLEX14SNP method (*9*). Presence of each genotype in the CAZ or ZAP regions is indicated. CAZ, ceftriaxone via intramuscular injection and oral azithromycin; ZAP, azithromycin, amoxicillin, probenecid.

Of the 717 samples that we subjected to iPLEX14SNP genotyping, 456 (63.6%) were successfully genotyped and 261 (36.4%) were not characterized. We excluded the uncharacterized samples from further analysis. We observed 27 different iPLEX14SNP genotypes for the 456 characterized samples and summarized the 14 most common (NG01 to NG14, consisting of those with 5 or more isolates; total, 437 isolates), indicating whether they were from the CAZ or ZAP regions (Figure 2). A total of 10 iPLEX14SNP genotypes were shared across both CAZ and ZAP regions, including 8 of the 14 most common. Examination of available PCR data associated with these 10 genotypes shared between CAZ and ZAP regions revealed that 3 possessed resistance markers, including NG07 (GyrA S91F, *mtrR* A-deletion, and PorB mutant sequences), NG13 (GyrA S91F and PPNG), and a third genotype (GyrA S91F *mtrR* A-deletion and PPNG), listed among “all others” in Figure 2.

### NG-MAST Investigation of mosaicPBP2-PCR Positive Samples

The NG-MAST analyses for the 10 samples positive by the mosaicPBP2-PCR (Table 3) revealed 4 different types, including 3 types (2212, 5622, and 1407) that have previously been associated with the gonococcal mosaic PBP2 in Europe and elsewhere (*21*). Additional demographic data were obtained; the 10 samples were from 7 men and 2 women, including 3 Aboriginal (1 man and 2 women) and 6 non-Aboriginal persons. All of these persons were identified in the metropolitan area of Darwin in the Northern Territory.

**Table 3:** Summary of mosaicPBP2-PCR–positive *Neisseria gonorrhoeae* samples from the Northern Territory of Australia*

Patient no.	Sex	Age group, y	Aborigine	Sample source	Date of collection	NG-MAST genotype
1	M	51–55	No	Urine	January 2014	10038
2	M	21–25	No	Urine	March 2014	2212
3	F	26–30	Yes	Urine	April 2014	2212
				Pharyngeal swab	May 2014	2212
4	M	26–30	Yes	Urine	April 2014	2212
5	F	<20	Yes	Urine	May 2014	2212
6	M	21–25	No	Urine	February 2014	5622
7	M	56–60	No	Urine	December 2014	5622
8	M	21–25	No	Anal swab	November 2014	1407
9	M	21–25	No	Anal swab	November 2014	1407

*NG-MAST, *N. gonorrhoeae* multiantigen sequence typing.

## Discussion

The results of this study show conclusively that molecular methods can be used to enhance *N. gonorrhoeae* AMR surveillance for a range of antimicrobial drugs in an isolated region where bacterial culture is impractical or not possible. For the Northern Territory of Australia, these new data substantially increase the evidence base for the current treatment guidelines, characterizing approximately two thirds of all notified cases, compared with only 13% (222/1740) that were available for characterization by culture. Furthermore, the data indicate no ceftriaxone resistance and little azithromycin resistance (0.2%) in this region and provide an estimate that penicillin resistance is <5% in the ZAP regions.

This case study also highlights the potential for molecular assays to inform alternative treatment strategies in areas where culture-based testing may be limited. The high ciprofloxacin susceptibility levels indicate that ciprofloxacin would be suitable as an alternative oral therapy in the ZAP regions; the GyrA91-PCR showed that 99.0% of infections in the ZAP regions were ciprofloxacin susceptible. Thus, ciprofloxacin could be used empirically or could otherwise be of value where use of the ZAP pack is contraindicated (e.g., penicillin allergy) or to facilitate patient-delivered partner therapy (PDPT). Although PDPT for chlamydia is supported in the Northern Territory, it is not recommended for gonorrhea given the risk of anaphylaxis with penicillin and the injection requirement for ceftriaxone. Based on our data, ciprofloxacin could appropriately be used for PDPT for gonorrhea in the ZAP regions, ideally in combination with azithromycin to simultaneously treat both chlamydia and gonorrhea given the high coinfection rates in these areas (*22*). 

A further benefit of using ciprofloxacin in ZAP regions is that rates of susceptibility/resistance could be monitored accurately by molecular methods, such as the GyrA91-PCR. This assay could also facilitate use of oral ciprofloxacin in the CAZ regions through individualized treatment, which, based on the prevalence of wild-type (susceptible) strains, could be used for 79.1% of patients, substantially sparing the use of ceftriaxone. Similarly, based on the combined results of the PorB-PCR and PPNG-PCR methods, individualized ZAP pack treatment could potentially be used for 73.5% of infections in the CAZ regions.

The data also provide some evidence of new threats to current treatments. Although there was no further evidence of the A8806 ceftriaxone-resistant strain, the detection of mosaicPBP2 strains in the Northern Territory, particularly among Aboriginal patients, is alarming. These mosaicPBP2 strains typically exhibit CMRP and therefore pose a direct risk to the use of ZAP packs. This risk is heightened by the ability of these mosaicPBP2 strains to spread rapidly. First reported in Japan in 2001, mosaic PBP2 strains have since become an internationally successful clone. Accordingly, our nationwide study of gonococcal isolates from Australia in 2012 showed that mosaic strains composed 8.9% of all isolates and had spread to every state in Australia except the Northern Territory (*23*). The NG-MAST data from this study (Table 3) indicated that different incursions of the mosaicPBP2 strains into the Northern Territory population occurred in 2014. It is likely that 3 of these involved men who have sex with men, residing in the metropolitan area of Darwin. The presence of the same NG-MAST type (2212; Table 3) in 1 Aboriginal man and 2 Aboriginal women strongly suggests transmission within heterosexual Aboriginal networks in the Darwin region.

It was previously assumed that penicillin resistance has remained low in ZAP regions because of limited bridging of sexual networks in metropolitan areas. This hypothesis was not supported by our MLST data, however, which showed that 10 of the 27 observed gonococcal strain types (including 8 of the 14 most common strain types; Figure 2) were present in both CAZ and ZAP regions. These strains included 3 strain types predicted to be resistant to ciprofloxacin, penicillin, or both, suggesting common sexual networks. Thus, it is theoretically possible that mosaicPBP2 strains or other resistant strains could exploit this bridging and spread into ZAP regions.

The addition of molecular strategies in the remote regions provided a notable increase in the scope of AMR surveillance. With the increased surveillance capture, the rates of both ciprofloxacin resistance and PPNG predicted by PCR were much lower (less than half) than rates detected by bacterial culture. In total, 9 times more samples from the ZAP regions were ascertained by PCR than by bacterial culture, compared with 4 times more samples from CAZ regions ascertained by PCR than by culture. Previous Australia data have shown that the GyrA91-PCR assay is an accurate predictor of ciprofloxacin resistance and the PPNG-PCR assay is an accurate predictor of PPNG (*11**,**14**,**15*). Therefore, it is unlikely that the observed differences between PCR and culture-based data were attributable to problems with accuracy of the PCR target sequences. Rather, we suspect that the observed differences in resistance rates were influenced primarily by the higher proportion of infections that were able to be characterized in the ZAP region with these PCR assays. Our findings strongly support the CDC’s and WHO’s positions on the utility of molecular strategies to enhance surveillance (*1**,**2*). Rates of drug resistance are rising, and refining and rationalizing antimicrobial drug use at the individual patient level is a desirable stewardship strategy.

Our study has several limitations. The predictive value of the molecular markers used in this study are based on Australia sample banks, so they may not be representative of other regions, where different gonococcal strains may be circulating. This limitation is particularly relevant for the PorB-PCR PIA target, which, as previously described, does not specifically target a mechanism of resistance, yet is highly associated with penicillin susceptibility in local Australia gonococci (*17*). These issues highlight the importance of maintaining culture-based AMR testing for definitive AMR surveillance, as well as for providing ongoing validation material for the molecular methods. In addition, we observed that approximately one third of *N. gonorrhoeae* NAAT-positive clinical samples could not be characterized by 1 or more of the resistance assays; this finding is likely due to low DNA loads, as previously documented (*16*). The lack of characterization was particularly evident for pharyngeal samples, which likely reflects *N. gonorrhoeae* DNA loads being generally lower in the pharynx compared with other sites.

Overall, this study highlights the potential for molecular methods to enhance culture-based AMR surveillance programs by increasing sample size. These methods have provided, and continue to provide, new representative data to inform local treatment guidelines, identifying alternative treatment options and pinpointing new resistance threats. Molecular methods, such as those described here, offer new opportunities to improve *N. gonorrhoeae* AMR surveillance globally, particularly in remote regions.

Technical Appendix Additional details for study of antimicrobial drug resistance in *Neisseria gonorrhoeae* among patients in the Northern Territory of Australia, 2014.
