# Supplementary material for: Molecular Antimicrobial Resistance Surveillance for Neisseria gonorrhoeae, Northern Territory, Australia
Source: Emerg Infect Dis. 2017 Sep;23(9):1478–85. doi: 10.3201/eid2309.170427 (PMC5572890; doi:10.3201/eid2309.170427)
Supplement: Technical Appendix — Additional details for study of antimicrobial drug resistance in Neisseria gonorrhoeae among patients in the Northern Territory of Australia, 2014. [file 17-0427-Techapp-s1.pdf]

Article DOI: https://doi.org/10.3201/eid2309.170427

Molecular Antimicrobial Resistance Surveillance for *Neisseria gonorrhoeae*, Northern Territory, Australia

Technical Appendix

Materials and Methods

DNA Extraction

Specimens received from Royal Darwin Hospital Pathology consisted of remnant DNA extracts from the Siemens Versant CT/NG assay. These DNA extracts were directly applied to the methods used in this study. Specimens received from Western Diagnostic Pathology consisted of original samples suspended in Aptima specimen transport medium. These samples needed to undergo DNA extraction before testing. We extracted a 200 µL aliquot of each suspension by using the Roche High Pure Viral Nucleic Acid kit (Roche Diagnostics, North Ryde, New South Wales, Australia) according to manufacturer's instructions.

PPNG-PCR

We used 2 versions of the PPNG-PCR. We tested the first 481 samples using our originally described PPNG-PCR (*1*). We tested the remaining samples using a modified PPNG-PCR (*2*), which became available during the testing and incorporates an additional sequence target to account for a rare plasmid variant (*3*).

GyrA91-PCR

The GyrA91-PCR included 2 probes, 1 for detection of the wild-type S91 sequence, and the other for detection of the S91F alteration. The assay was performed as previously described (*4*), with the exception that alternative primers (NG-GYRASER91-F and R), described previously, were used (*5*). We have subsequently shown that the *N. gonorrhoeae* GyrA S91F alteration is highly predictive of ciprofloxacin resistance (≈99% agreement with bacterial culture (*6*)).

23S rRNA 2611-PCR and 2059-PCR Assays

We performed the real-time PCR assays, 2611-PCR and 2059-PCR, as described previously (*7*). These assays targeted the gonococcal 23S rRNA C2611T and A2059G mutations, 2 *N. gonorrhoeae* 23S rRNA mutations commonly associated with azithromycin resistance.

PorB-PCR

We performed the PorB-PCR assay as previously described (*8*), and used 6 probes to characterize various combinations of amino acids at positions 101 and 102 of the PorB1b class protein, including the wild-type G101/A102 and mutant G101K/A102D, G101K/A102N and G101K/A102G sequences, as well as the PorB1a sequence. When used in parallel with the previously described PPNG-PCR to exclude PPNG, the PorB-PCR can be used to predict susceptibility to penicillin. Briefly, previous validation data (*8*) are summarized as follows: testing of 2,307 *N. gonorrhoeae* isolates from throughout Australia showed that the PorB-PCR G101/A102 and PorB1a sequences provided positive predictive values of 98.8% and 98.9% for penicillin susceptibility (where PPNG strains were simultaneously identified and excluded); however, this testing could identify only 78.4% of all penicillin-susceptible isolates. The mutant sequences were highly associated with chromosomally mediated resistance to penicillin (CMRP) ($p < 0.001$), but not sufficiently predictive of CMRP (e.g., ≈40% of isolates with mutant sequences were penicillin susceptible).

mtrR A-deletion-PCR Assay

We performed the A-deletion-PCR assay as described previously (*9*). Briefly, this assay uses an allelic discrimination approach to distinguish *mtrR* wild-type isolates from those that have an A-deletion in the *mtrR* promoter region. This *mtrR* A-deletion results in increased efflux pump activity and is associated with gonococcal resistance to several classes of antimicrobial drugs, including β lactam antimicrobials. Although it cannot be used to accurately predict resistance to any antimicrobial drug, the wild-type *mtrR* promoter is highly associated with susceptibility to β-lactams; our analysis of 2,380 isolates from throughput Australia from 2012 showed that only 0.5% (8/1,728) of isolates with the wild-type promoter exhibited decreased susceptibility to ceftriaxone (CRO-DS), whereas 13.0% (85/652) with the A-deletion were CRO-DS ($p < 0.001$). Likewise, for penicillin (excluding 353 PPNG isolates), only 4.4% (62/1,425) of wild-type isolates exhibited CMRP, whereas 59.8% (360/602) of A-deletion isolates were CMRP ($p < 0.001$) (unpub. data).

MosaicPBP2-PCR Assay

Emerging *N. gonorrhoeae* resistance to the extended-spectrum cephalosporins is associated principally with a mosaic penicillin-binding protein 2 (PBP2) (*10*). Strains harboring a mosaic PBP2 typically exhibit resistance to cefixime, CRO-DS, CMRP, and both ciprofloxacin and tetracycline. The previously documented ceftriaxone resistant strains, including the H041, F89 and A8806 strains, all possessed variants of the mosaic PBP2 protein. The mosaicPBP2-PCR assay was designed as part of this study, based on a previously described method (*11*), to enable detection of previously described mosaic-PBP2 harboring strains, including the previously mentioned ceftriaxone resistant strains, but not other gonococci lacking a mosaic PBP2. Each reaction mix consisted of 10.0 µL of QuantiTect Probe PCR master mix (Qiagen, Melbourne, Victoria, Australia); 10.0 µM each of forward and reverse primer (GTATTGCGGGATCGGGAA and CCRATTTTGTAAGGCARGGT, respectively); 0.2 µM probe (FAM-TTACGCGGAGAATCCAAACTGTCCA-BHQ); and 2.0 µL of nucleic acid extract or control, and were made up to a final reaction volume of 20.0 µL using PCR-grade water. We performed amplification and detection using both the Applied Biosystems ViiA 7 (Life Technologies, Scoresby, Victoria, Australia) and Rotor-Gene Q real-time PCR (Qiagen) instruments using the following cycling parameters: an initial hold at 95°C for 15 min, followed by 45 cycles at 95°C for 15 s and then 60°C for 60 seconds. For the mosaicPBP2-PCR (and A8806-PCR), we pooled samples (10 samples per pool) for testing; samples from any pools returning positive results were then tested individually. For all other PCR methods, we tested samples individually.

A8806-PCR Assay

We designed this assay as part of this study to target a combination of sequences unique to A8806: the Australian ceftriaxone-resistant strain (*12*). We performed this assay as we did the mosaicPBP2-PCR, except that we used a different reverse primer (AGGGTATTGAATGTGTCTGTTGGA) and probe (FAM-ATCCCAACAGACCCGGCCG-BHQ). As described earlier, we used pooled samples for screening for A8806.

Results

Results of the assays are given in Table 1 in the main article.

A comparison of characterization rates based on anatomic site and originating laboratory is given in the Technical Appendix Table; only the GyrA91-PCR results were used for this comparison.

References

1. Goire N, Freeman K, Tapsall JW, Lambert SB, Nissen MD, Sloots TP, et al. Enhancing gonococcal antimicrobial resistance surveillance: a real-time PCR assay for detection of penicillinase-producing *Neisseria gonorrhoeae* by use of noncultured clinical samples. J Clin Microbiol. 2011;49:513–8. http://dx.doi.org/10.1128/JCM.02024-10

2. Buckley C, Trembizki E, Baird RW, Chen M, Donovan B, Freeman K, et al. Multitarget PCR assay for direct detection of penicillinase-producing *Neisseria gonorrhoeae* for enhanced surveillance of gonococcal antimicrobial resistance. J Clin Microbiol. 2015;53:2706–8. http://dx.doi.org/10.1128/JCM.00540-15

3. Trembizki E, Buckley C, Lawrence A, Lahra M, Whiley D; GRAND Study Investigators. Characterization of a novel *Neisseria gonorrhoeae* penicillinase-producing plasmid isolated in Australia in 2012. Antimicrob Agents Chemother. 2014;58:4984–5. http://dx.doi.org/10.1128/AAC.02993-14

4. Buckley C, Trembizki E, Donovan B, Chen M, Freeman K, Guy R; GRAND Study Investigators. A real-time PCR assay for direct characterization of the *Neisseria gonorrhoeae* GyrA 91 locus associated with ciprofloxacin susceptibility. J Antimicrob Chemother. 2016;71:353–6. http://dx.doi.org/10.1093/jac/dkv366

5. Siedner MJ, Pandori M, Castro L, Barry P, Whittington WL, Liska S, et al. Real-time PCR assay for detection of quinolone-resistant *Neisseria gonorrhoeae* in urine samples. J Clin Microbiol. 2007;45:1250–4. http://dx.doi.org/10.1128/JCM.01909-06

6. Trembizki E, Guy R, Donovan B, Kaldor JM, Lahra MM, Whiley DM; GRAND study investigators. Further evidence to support the individualised treatment of gonorrhoea with ciprofloxacin. Lancet Infect Dis. 2016;16:1005–6. http://dx.doi.org/10.1016/S1473-3099(16)30271-7

7. Trembizki E, Buckley C, Donovan B, Chen M, Guy R, Kaldor J, et al. Direct real-time PCR-based detection of *Neisseria gonorrhoeae* 23S rRNA mutations associated with azithromycin resistance. J Antimicrob Chemother. 2015;70:3244–9.

8. Buckley C, Trembizki E, Donovan B, Chen M, Freeman K, Guy R, et al.; Gonorrhoea Resistance Assessment by Nucleic Acid Detection (GRAND) Study Investigators. Real-time PCR detection of *Neisseria gonorrhoeae* susceptibility to penicillin. J Antimicrob Chemother. 2016;71:3090–5. http://dx.doi.org/10.1093/jac/dkw291

9. Goire N, Freeman K, Lambert SB, Nimmo GR, Limnios AE, Lahra MM, et al. The influence of target population on nonculture-based detection of markers of *Neisseria gonorrhoeae* antimicrobial resistance. Sex Health. 2012;9:422–9. http://dx.doi.org/10.1071/SH12026

10. Unemo M, Nicholas RA. Emergence of multidrug-resistant, extensively drug-resistant and untreatable gonorrhea. Future Microbiol. 2012;7:1401–22. http://dx.doi.org/10.2217/fmb.12.117

11. Ochiai S, Ishiko H, Yasuda M, Deguchi T. Rapid detection of the mosaic structure of the *Neisseria gonorrhoeae* penA Gene, which is associated with decreased susceptibilities to oral cephalosporins. J Clin Microbiol. 2008;46:1804–10. http://dx.doi.org/10.1128/JCM.01800-07

12. Lahra MM, Ryder N, Whiley DM. A new multidrug-resistant strain of *Neisseria gonorrhoeae* in Australia. N Engl J Med. 2014;371:1850–1. http://dx.doi.org/10.1056/NEJMc1408109

Technical Appendix Table. Comparison of GyrA91-PCR characterization rates based on anatomic site and originating laboratory, Australia, 2014

	Royal Darwin Hospital Pathology* n = 815; 446 from ZAP and 369 from CAZ regions			Western Diagnostic Pathology† n = 814; 742 from ZAP and 72 from CAZ regions			Total n = 1629; 1,188 from ZAP and 441 from CAZ regions		
Anatomic site	No. samples	C‡	UC‡	No. samples	C	UC	No. samples	C	UC
Cervical/ vaginal	232	155 (66.8%)	77 (33.2%)	181	153 (84.5%)	28 (15.5%)	413	308 (74.6%)	105 (25.4%)
Joint fluid	21	16 (76.2%)	5 (23.8%)	0	0	0	21	16 (76.2%)	5 (23.8%)
Not assigned	14	10 (71.4%)	4 (28.6%)	5	5 (100%)	0	19	15 (79%)	4 (21%)
Ocular	1	1 (100%)	0	0	0	0	1	1 (100%)	0
Pharyngeal	44	11 (25%)	33 (75%)	1	1 (100%)	0	45	12 (26.7%)	33 (73.3%)
Rectal	21	12 (57.1%)	9 (42.9%)	0	0	0	21	12 (57.1%)	9 (42.9%)
Urethral	71	64 (90.1%)	7 (9.9%)	26	26 (100%)	0	97	90 (92.8%)	7 (7.2%)
Urine	411	267 (65%)	144 (35%)	601	507 (84.4%)	94 (15.6%)	1012	774 (76.5%)	238 (23.5%)
Totals	815	536 (65.8%)	279 (34.2%)	814	692 (85%)	122 (15%)	1629	1228 (75.4%)	401 (24.6%)

*Samples consisting of those providing *Neisseria gonorrhoeae* (NG) positive results by the Siemens Versant CT/NG assay.
†Samples consisting of those providing NG positive results by the Aptima Combo 2 CT/NG assay.
‡C, characterized; UC, uncharacterized.
